# Ten years preceding a diagnosis of neurodegenerative disease in Europe and Australia: medication use, health conditions, and biomarkers associated with Alzheimer's disease, Parkinson's disease, and amyotrophic lateral sclerosis

**DOI:** 10.1016/j.ebiom.2025.105585

**Published:** 2025-02-05

**Authors:** Dang Wei, Anna Freydenzon, Octave Guinebretiere, Karim Zaidi, Fen Yang, Weimin Ye, Niklas Hammar, Karin Modig, Naomi R. Wray, Maria Feychting, Nadine Hamieh, Bruno Ventelou, Beranger Lekens, Laurene Gantzer, Stanley Durrleman, Allan McRae, Baptiste Couvy-Duchesne, Fang Fang, Thomas Nedelec, LeMeReND consortium, Stanley Durrleman, Bruno Ventelou, Thomas Nedelec, Octave Guinebretiere, Karim Zaidi, Fang Fang, Dang Wei, Fen Yang, Allan McRae, Naomi Wray, Baptiste Couvy-Duchesne, Anna Freydenzon, Lydie Tran, Evans Cheruiyot

**Affiliations:** a Institute of Environmental Medicine, Karolinska Institutet, Stockholm, Sweden; b Institute for Molecular Biosciences, The University of Queensland, Brisbane, Australia; c Sorbonne University, Paris Brain Institute – ICM, CNRS, Inria, Inserm, AP-HP, Hôpital de la Pitié Salpêtrière, Paris, France; d Department of Epidemiology and Biostatistics, Karolinska Institutet, Stockholm, Sweden; e Department of Psychiatry, University of Oxford, Oxford, UK; f Aix-Marseille University, CNRS, IRD, AMSE, Marseille, France; g Cegedim R&D, Boulogne-Billancourt, France

**Keywords:** Neurodegenerative disease, Alzheimer's disease, Parkinson's disease, Amyotrophic lateral sclerosis, Medications, Health conditions, Biomarkers

## Abstract

**Background:**

Many studies have investigated early predictors for Alzheimer's disease (AD), Parkinson's disease (PD), and amyotrophic lateral sclerosis (ALS). However, evidence is sparse regarding specific and common predictors for these diseases. We aimed to identify medication use, health conditions, and blood biomarkers that might be associated with the risk of AD, PD, and ALS ten years later.

**Methods:**

We conducted population-based nested case–control studies of AD, PD, and ALS using electronic medical records in Europe (France, the UK, and Sweden) and Australia. We retrieved data on medication use, diagnosed health conditions, and measured blood biomarkers from electronic medical records or biomedical cohorts. Conditional logistic regression models and meta-analysis were applied to assess the associations between these factors and the risk of receiving a diagnosis of AD, PD, or ALS.

**Findings:**

We included a total of 149,642 AD cases (mean age: 79.1–81.2 years), 252,696 PD cases (73.2–75.9 years), and 27,533 ALS cases (64.4–69.6 years). The prescription of psychoanaleptics and nasal preparations was consistently associated with an increased risk of AD, PD, and ALS 5–10 years later. Constipation and use of related medications were associated with an increased risk of AD and PD, while diabetes and use of antidiabetics were associated with a reduced risk of ALS. A higher level of triglycerides was associated with a lower risk of AD, whereas a higher level of Apolipoprotein B was associated with a lower risk of PD, 5–10 years later.

**Interpretation:**

Psychoanaleptics and nasal preparations may serve as common predictors for diagnosis of AD, PD, and ALS 5–10 years later. Conversely, the increased prevalence of constipation is specific to AD and PD, while the decreased prevalence of diabetes and use of antidiabetics is specific to ALS.

**Funding:**

EU Joint Programme—Neurodegenerative Disease Research.

**Research in context**

**Evidence before this study**

We searched PubMed from inception until May 30, 2024 for English original articles using the following search strategy: (“dementia” [Title/Abstract] OR “alzheimer” [Title/Abstract] OR “parkinson” [Title/Abstract] OR “amyotrophic lateral sclerosis” [Title/Abstract] OR “ALS” [Title/Abstract]) AND (“prodromal” [Title/Abstract] OR “prediagnostic” [Title/Abstract] OR “pre-diagnostic” [Title/Abstract]). We screened for studies with a focus on medications, health conditions, and blood biomarkers during the prodromal stage (up to 20 years) of the three neurodegenerative diseases (i.e., Alzheimer's disease [AD] and other dementias, Parkinson's disease [PD], and amyotrophic lateral sclerosis [ALS]) and found 18 studies (6 in AD and other dementias, 9 in PD, 2 in ALS, and 1 in both AD and PD). Most of the studies focused on one factor or one group of factors. There are six data-driven studies that evaluated medications and health conditions before the diagnosis of AD and other dementias or PD. Briefly, these studies identified several common factors that are associated with the risk of AD and other dementias, and PD 0–2 or 2–10 years later, including depression, anxiety, constipation, sleep issues, memory loss, fatigue, and low body weight. In contrast, some prodromal factors were only observed for specific diseases, e.g., stress-related disorders, hypertension, arthritis, vision impairment, coronary heart disease, stroke, diabetes, loneliness, and benzodiazepines use, etc., for AD and other dementias; fracture, hypotension, and use of antibiotics, psychoanaleptics, medicines for constipation, urologicals, etc., for PD; and a high level of plasma neurofilament light chain for ALS. Previous studies utilised various data sources (e.g., established cohorts, national registries) and focused on different timeframes prior to diagnosis, leading to a challenge in directly comparing results across studies. Further, none of the studies has attempted to identify common prodromal factors, including medications, health conditions, and blood biomarkers, across the three neurodegenerative diseases.

**Added value of this study**

The study leverages data from Australia, France, the UK, and Sweden with the aim to identify medications, health conditions, and blood biomarkers associated with the risk for AD, PD, and ALS during the following years (i.e., 2–5 and 5–10 years prior to diagnosis). Meta-analyses were conducted for 49 medications and 73 health conditions in AD, 45 medications and 134 health conditions in PD, and 43 medications and 131 health conditions in ALS. Psychoanaleptics and nasal preparations were found to be consistently associated with an increased risk of AD, PD, and ALS 5–10 years later. Several indications for psychoanaleptics, e.g., depression, anxiety, sleep disorders, etc., were also associated with the risk of AD and PD 5–10 years later. Similar results were exhibited for constipation and use of related medications in relation to AD and PD. Finally, diabetes and use of antidiabetics were found to be consistently associated with a lower future risk of ALS.

**Implications of all the available evidence**

Together with the existing knowledgebase, this work adds on important evidence for identifying individuals at high risk of developing neurodegenerative diseases using information on medication use, diagnosed health conditions, and blood biomarkers. It also provides potential targets for impeding the progression or postponing the onset of these neurodegenerative diseases.

## Introduction

Neurodegenerative diseases (NDDs) pose a significant public health challenge due to their rising prevalence among ageing populations and substantial healthcare expenditure.^1^, ^2^, ^3^ Alzheimer's disease (AD) and other dementias, affecting 52 million people in 2019,^2^ are estimated to impact 150 million in 2050, worldwide.^4^ Parkinson's disease (PD), the second most common NDD, affected 6.1 million people in 2016, representing a 145% increase since 1990.^3^ Motor neuron disease, with amyotrophic lateral sclerosis (ALS) as the major type, is also on the rise, affecting about 160,000 individuals in 1990 and 269,000 in 2019.^5^

Several factors, including depression, anxiety, constipation, sleep issues, memory loss, fatigue, and low body weight, have been found to be associated with an increased risk of AD and other dementias and PD up to 10 years later in previous studies.^6^, ^7^, ^8^, ^9^ However, there is a challenge to compare findings across studies as they used various data sources and focused on different timeframes prior to the diagnosis of NDDs and none has attempted to evaluate factors commonly associated with several NDDs. It also remains unclear whether prescribed medications and blood biomarkers could be commonly predictive of future NDDs and whether the results are consistent across populations with different healthcare systems.

To this end, we set up an international consortium utilising electronic medical records and biomedical cohort data from Australia, France, the UK, and Sweden, covering a common timeframe up to 10 years before the diagnosis of NDDs. We systematically evaluated a series of prescribed medications, clinically diagnosed health conditions, and blood biomarkers before the diagnosis of AD, PD, and ALS. We sought to identify a comprehensive list of potential predictors for future NDDs, which are either specific or generic to different NDDs and applicable to various populations.

## Methods

### Data sources and study design

#### Australia

We investigated the associations of prescribed medications with subsequent risk of ALS using data from the Australian Institute of Health and Wellness (AIHW) which is an independent statutory agency that collects, analyses, and disseminates Australian health and welfare data, including performing linkage across national data collections, particularly the National Death Index (NDI) and Pharmaceutical Benefits Scheme (PBS). The NDI consists of all records of death in Australia since 1980, with information on the date and the main and secondary causes of death.^10^ The PBS data collection, started in 1984, meticulously records prescriptions eligible for benefits, encompassing information on drug categorisation, cost, the prescriber, and the dispensing pharmacy.^11^ An extraction fee was required to use data from AIHW.

ALS cases were initially identified through the underlying cause of death recorded in the NDI and a prescription of Riluzole in the PBS (Table S1). The NDI and PBS data extraction spanned from the beginning of data collection (January 1, 1997 for NDI and July 1, 2002 for PBS), to December 31, 2018. We used the date of first Riluzole prescription as a proxy for diagnosis date of ALS. We excluded cases identified from NDI only and those diagnosed before July 1, 2004 (with <2 years of time for identifying prescribed medications). We randomly selected 10 controls for each case who were alive and free of ALS on the diagnosis date of the case, and individually matched the controls to the case by sex and birth date (±15 days), leading to a nested case–control study of 6621 ALS cases and 66,210 matched controls (Figure S1). Date of diagnosis of the case was defined as the index date of the case and their individually matched controls. We then identified prescribed medications prior to the index date, using the first three digits of the Anatomical Therapeutic Chemical (ATC) codes, from PBS for both the cases and controls.

#### France: SNDS

We investigated the associations of prescribed medications and health conditions with subsequent risk of PD and ALS using data from the French National Health Data System (SNDS). SNDS is a comprehensive and extensive repository of healthcare information established in 2009 with the aim to catalogue all reimbursements from public health insurance for all residents in France. The repository incorporates data from two primary sources: the national hospital discharge database for inpatient care and the national health insurance claims information system for outpatient care, covering approximately 99% of the population of France. SNDS contains detailed information on all reimbursed prescriptions coded by ATC codes in both inpatient and outpatient services. SNDS also includes information on clinical diagnoses from hospitalisation records and long-term disease (LTD) scheme, i.e., LTD listed (and reimbursed) specifically to alleviate the financial hardship. To access and process data from the SNDS, permanent access to the CNAM (Caisse Nationale de l'Assurance Maladie - French National Health Insurance Fund) data portal was granted via the INRIA (Institut National de Recherche en Informatique et en Automatique - French National Institute for Research in Digital Science and Technology) affiliation of the ARAMIS (Algorithms, models and methods for images and signals of the human brain) team, in application of the provisions of Articles R. 1461–11 to R. 1461-17 of the French Public Health Code and the French data protection authority (CNIL) decision CNIL-2016-316.

We identified PD and ALS cases using validated case-finding algorithms incorporating information on specific prescribed medications (ATC codes: N04A or N04B [anti-parkinsonism drugs] for PD; N07XX02 [Riluzole] for ALS), hospital discharge diagnoses, and diagnosis codes related to benefits from the LTD scheme (Table S1).^12^^,^^13^ To ensure a sufficient time-window to study prescribed medications and health conditions before the diagnosis of PD and ALS, we included only cases diagnosed between 2014 and 2021, leading to a total of 195,575 PD cases and 14,056 ALS cases (Figure S2). Date of diagnosis was determined as the earliest occurrence among the prescription of the specific medications, a hospital discharge diagnosis of PD or ALS, or the inclusion in the LTD scheme for PD or ALS. For each PD and ALS case, we randomly selected one and ten controls who had records for other conditions but PD and ALS in the SNDS, respectively. The choice of one rather than ten controls for PD was due to practical and computational considerations. Cases and controls were individually matched based on sex, year of birth, and French regional code of residence. For all cases and controls, we extracted ATC codes of prescribed medications recorded in outpatient care data, as well as the 10th version of International Classification of Diseases (ICD-10) codes for health conditions recorded in hospitalisation discharge and LTD benefits records from 2009 until the index date. Prescribed medications in inpatient care data were however not included.

#### France and the UK: THIN

We investigated the associations of prescribed medications and health conditions with subsequent risk of AD using data from The Health Improvement Network (THIN) database that contains primary care data from France and the UK.^14^ The THIN database contains information on prescribed medications and clinical diagnoses from 2500 general practitioners in France and 532 general practitioners in the UK, including 8 million patients in total. The French data are representative of the French general population in terms of age, sex, and residence.^15^ The UK data, accounting for around 6% of the UK population, accurately reflect the demographics and types of consultation within the entire UK population.^16^ The data are available from the Cegedim company upon request for researchers who meet the criteria for access to confidential data.

We identified AD cases diagnosed between January 4, 1998 and February 20, 2019 in France, and between January 1, 1996 and March 31, 2020 in the UK from the THIN database, leading to a total of 19,458 cases from France and 20,214 cases from the UK (Figure S3). One control was randomly selected and individually matched to each case based on sex and age at the last contact with general practitioners as previously reported.^9^ We converted Read codes version 3 used in the UK to ICD-10 codes following the structure provided by the UK Clinical Terminology Centre. ICD-10 codes were directly available in the French THIN database. We then identified medications by ATC codes and health conditions by ICD-10 codes recorded in the THIN database before the index date for both the cases and controls (Table S1).

#### Sweden

We studied the associations of prescribed medications and health conditions with subsequent risk of AD, PD, and ALS using data from several Swedish national population and health registers. First, we defined a cohort of 13,455,011 individuals registered in the Swedish Total Population Register during 1961–2016. We identified those with a diagnosis of NDDs based on the primary or secondary diagnoses from inpatient (started in 1964 and became nationwide in 1987) or outpatient (since 2001) care recorded in the Swedish Patient Register, using ICD codes (Table S1).^17^ We defined the study period for the analysis of health conditions as 1987–2016 because the Patient Register became nationwide in 1987. After excluding individuals with a diagnosis of any NDD (dementias, PD, Parkinsonism, ALS or other motor neuron diseases, or other movement or degenerative disorders) before 1987, we identified 109,881 cases of AD, 57,121 cases of PD, and 6847 cases of ALS (Figure S4). We defined the date of first hospital contact concerning the NDDs as the diagnosis date of an NDD (i.e., index date) and randomly selected ten controls per case of NDD from the Swedish Total Population Register, who were free of any NDDs at the index date and individually matched to the case by sex and year of birth, using the method of incidence density sampling. We identified all clinically diagnosed health conditions made at an inpatient or outpatient hospital visit before the index date through the Swedish Patient Register, using ICD codes for both the cases and controls. We grouped the diagnosed health conditions with clinical or biological similarities (Tables S2–S6).

Since July 2005, the Swedish Prescribed Drug Register has collected data on filled prescriptions of medications in all Swedish pharmacies.^18^ We similarly conducted three nested case–control studies with a study period of 2007–2016 to examine the associations of prescribed medications with subsequent risk of NDDs, including 39,341 cases of AD, 18,209 cases of PD, and 3062 cases of ALS, as well as their individually matched controls, respectively. We identified all prescribed medications before the index date through the Prescribed Drug Register, using ATC codes.

We assessed the associations of blood biomarkers with subsequent risk of NDDs using data from the Swedish Apolipoprotein-related MOrtality RISk (AMORIS) cohort. AMORIS is a population-based cohort including 812,073 individuals undergoing health examinations during 1985–1996 who mainly resided in the Stockholm region. The health examinations included the collection of blood or urine sample from either a routine occupational health check-up or through outpatient care.^19^ Details of the AMORIS cohort are reported elsewhere.^19^ We limited our analysis to four groups of biomarkers measured in blood, namely lipid, apolipoprotein, glucose, and inflammatory biomarkers, which were available for at least 20% of individuals included in the cohort. As described above, we identified the first diagnosis of AD, PD, and ALS among individuals included in the AMORIS cohort by linking to the Swedish Patient Register with data available until 2020. We excluded individuals whose blood biomarkers were measured after the diagnosis and those with incorrect information on birth year, resulting in 22,294 AD cases, 9000 PD cases, and 1215 ALS cases in the analysis (Figure S5). Similarly, we randomly selected 10 controls per case, individually matched by sex and birth year, as controls for the cases using incidence density sampling methods.

The Swedish population and health registers are kept by different Swedish authorities (e.g., the Swedish National Board of Health and Welfare and Statistics Sweden) for administrative purposes and can be used for research purposes after permission from the Swedish Ethical Review Authority and a legal assessment by the registry holders.

**Fig. 1:**
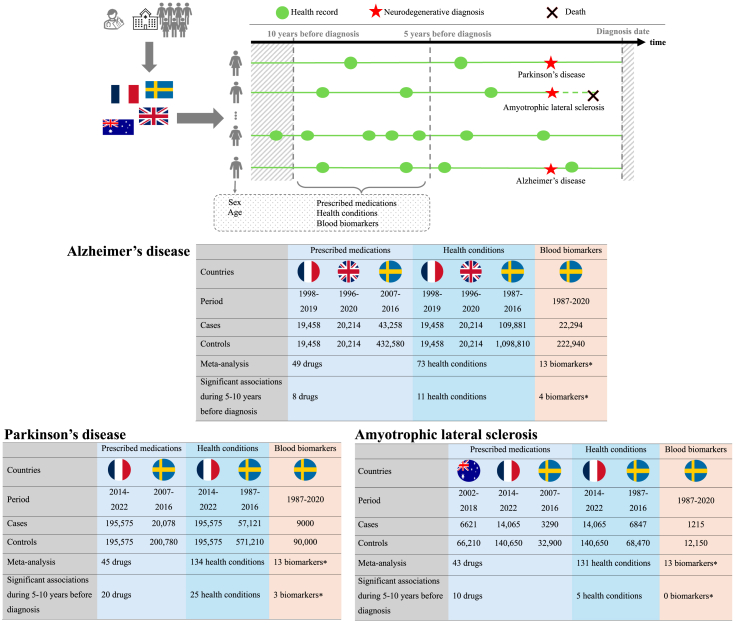
**Summary of study design and results for investigating the associations of prescribed medications, health conditions, and blood biomarkers with the subsequent diagnosis of Alzheimer's disease, Parkinson's disease, and amyotrophic lateral sclerosis in Australia, France, the UK, and Sweden**. ∗No meta-analysis was performed since the analysis of blood biomarkes was only available in Sweden. As blood biomarkers were measure at midlife (mean: 43 years), we reported the results of biomarkers measured >10 years before diagnosis.

Our primary analysis focused on prescribed medications associated with NDDs using data from all four countries (AD: 79,013 cases and 433,082 controls; PD: 213,784 cases and 377,665 controls; ALS: 23,748 cases and 237,480 controls). In a secondary analysis, we evaluated whether there were clinically diagnosed health conditions associated with NDDs using data from France, the UK, and Sweden (AD: 149,553 cases and 1,138,483 controls; PD: 252,696 cases and 766,785 controls; ALS: 27,533 cases and 275,330 controls). Moreover, we investigated if biomarkers commonly measured in clinical chemistry were associated with NDDs in Sweden (AD: 22,294 cases and 222,940 controls; PD: 9000 cases and 90,000 controls; ALS: 1215 cases and 12,150 controls; Fig. 1).

As the first three digits of ATC codes were commonly available in our databases, we studied all families of prescribed medications identified by the first three digits. Eventually, there were 68 medications available for the analysis of ALS in Australia, 86 for AD, 67 for PD, and 65 for ALS in France, 84 for AD in the UK, as well as 55 for AD, PD, and ALS in Sweden. To limit the number of statistical tests, we selected health conditions that were recorded in >0.1% of the medical visits among cases of each NDD per 1000 person-years. Finally, we selected 98 health conditions for AD, 237 for PD, and 206 for ALS in France, 98 for AD in the UK, as well as 153 for AD, 160 for PD, and 175 for ALS in Sweden.

### Ethics

Written informed consent from study participants was not needed as we only utilised administrative data for analysis in this study. Further, no steps were taken for patient and public involvement in the research study. As permanent users of the SNDS, we declared the analyses in France and the UK to the INRIA's SNDS registry and were exempted from Institutional Review Board approval. The analyses in Australia and Sweden have been approved by the Australian Institute of Health and Wellness Ethics Committee (EO2019/1/481) and the Swedish Ethical Review Authority (2012/1814-31/4, and 2018/2401-31), respectively.

### Statistics

We calculated incidence rate ratios (IRR) and 95% confidence intervals (CI) for the associations of prescribed medications and health conditions with subsequent risk of AD, PD, and ALS in each country, using conditional logistic regression. As we applied nested case–control study design in which controls of a case were identified through incidence density sampling, the length of follow-up was identical for a case and their control(s) in a matched set. As a result, an odds ratio estimated from the conditional logistic regression can be interpreted as IRR. We disregarded the first two years before the index date to eliminate or diminish reverse causality, as the average diagnostic delay ranges from 1 to 7.5 years for NDDs.^20^, ^21^, ^22^ To further eliminate potential reverse causality, we analysed prescribed medications and health conditions for the periods of 2–5 and 5–10 years before the index date separately. We defined individuals as exposed when the studied prescribed medications and health conditions were identified at least once during the two periods separately. As we were mostly interested in 5–10 years before diagnosis of NDDs, we also performed the analysis for prescribed medications 5–10 years prior to the index date among men and women separately. Given that NDDs often have prodromal phases longer than a decade, we also analysed health conditions occurring >10 years before the index date in Sweden, where extensive data on health conditions were available for periods exceeding 10 years prior to diagnosis.

As participants of the AMORIS cohort were relatively young at the baseline health examination (mean: 43 years), we performed the analyses of blood biomarkers for the periods of 5–10 and >10 years before the index date separately. To assure sufficient statistical power, we also re-ran the analysis of blood biomarkers ≥2 years before the index date. Blood biomarkers were categorised according to sex-specific clinical cutoffs and the lower levels of the biomarkers were defined as the reference group. We adjusted for sex, country of birth, calendar year of blood sampling, calendar year of the index date, and age at the index date in the analysis of blood biomarkers. The covariates were considered as potential confounders if they were^1^ causes of changes in blood biomarkers or NDD or both,^2^ proxies for unmeasured common causes of changes in blood biomarkers and NDD, and^3^ not an instrumental variable.^23^

We meta-analysed the country-specific results of prescribed medications and health conditions using the DerSimonian and Laird method of random effect models and defined statistical significance as *P*-value <0.05/number of tests, corresponding to Bonferroni correction for multiple comparison. Confidence intervals were calculated according to the alpha level of 0.05/number of tests in two-tailed tests. To account for the potential bias in meta-analysis with few studies, we re-ran the meta-analysis for medications 5–10 years before diagnosis of AD, PD, and ALS using the Hartung-Knapp-Sidik-Jonkman method of random effect models.^24^^,^^25^

The meta-analyses were only performed for the medications and health conditions that were analysed in the specific countries. There were 49 families of medications available for the meta-analysis of AD from France, the UK, and Sweden, 45 for PD from France and Sweden, and 43 for ALS from Australia, France, and Sweden. We meta-analysed a total of 73 health conditions from France, the UK, and Sweden for AD, 134 from France and Sweden for PD, as well as 131 from France and Sweden for ALS.

Our previous study showed that patients with AD had a mean of seven visits to general practitioners per year before diagnosis.^9^ As we have data during at least seven and at most 38 years before diagnosis of NDDs in the study, we estimated that cases of NDDs might have from 49 to 266 visits to general practitioners before diagnosis in the present study. As we focused on a health condition that constituted at least 0.1% of all healthcare visits of the cases in 1000 person-years, the prevalence of a studied health condition should range from 4.9% to 26.6%. Further, as our data incorporate data from not only primary care but also outpatient and inpatient care, we assumed that the prevalence of a studied health condition would be around 10%. With such prevalence of an exposure, to detect an IRR of 1.20, i.e., the lowest average estimate for the associations between different health conditions and AD as demonstrated in our previous study,^9^ a minimum of 32,021 study participants was required to obtain 80% statistical power. This calculation accounts for multiple testing with approximately 50 medication families, 130 health conditions, three NDDs, and two distinct time periods (α = 0.05/(50 × 130 × 3 × 2) = 1.3 × 10⁻⁶). Notably, the sample size for each NDD in all countries exceeds this minimum threshold, ensuring robust statistical power.

### Role of funders

The founders had no role in study design, data collection, data analysis, data interpretation, or writing of the paper.

## Results

We included a total of 149,553 cases and 1,138,482 controls for the analysis of AD, 252,696 cases and 766,785 controls for PD, as well as 27,533 cases and 275,330 controls for ALS (Table 1). The mean ages at the diagnosis of AD and PD were similar across countries (79–81 and 73–76 years, respectively), while patients with ALS in Australia were diagnosed at a relatively younger age than in France and Sweden (64 vs. 68 vs. 70 years). Sex differences in the incidence of NDDs were consistent across countries, with a female predominance in AD but a male predominance in PD and ALS.

**Table 1:** Characteristics of individuals with Alzheimer's disease, Parkinson's disease, or amyotrophic lateral sclerosis and their individually matched controls in Australia, France, the UK, and Sweden.

Characteristics	Alzheimer's disease				Parkinson's disease			Amyotrophic lateral sclerosis			
	France	The UK	Sweden 1	Sweden 2	France	Sweden 1	Sweden 2	Australia	France	Sweden 1	Sweden 2
N of cases/controls^a^	19,056/19,056	19,940/19,940	109,881/1,098,810	39,341/393,410	195,575/195,575	57,121/571,210	18,209/182,090	6621/66,210	14,065/140,650	6847/68,470	3062/30,620
Age at diagnosis (years), mean (SD)	79.2 (9.8)	80.9 (8.4)	81.2 (8.0)	81.1 (8.3)	73.2 (9.7)	75.9 (9.7)	74.7 (10.4)	64.4 (11.9)	67.5 (12.5)	69.7 (12.1)	69.6 (12.2)
Sex, N (%)											
Men	6870 (36.1)	6016 (30.1)	40,686 (37.0)	15,181 (38.6)	111,223 (56.9)	32,630 (57.1)	10,850 (59.6)	3956 (59.7)	7803 (54.5)	3825 (55.9)	1686 (55.1)
Women	12,186 (63.9)	13,924 (69.8)	69,195 (63.0)	24,160 (61.4)	84,352 (43.1)	24,491 (42.9)	7359 (40.4)	2665 (40.3)	6262 (45.5)	3022 (44.1)	1376 (44.9)
Year of diagnosis, N (%)											
<1990	0	8 (0.4)	14,683 (13.4)	0	0	5677 (9.9)	0	0	0	469 (6.8)	0
1990–1999	19 (0.09)	1940 (9.7)	31,893 (29.0)	0	0	17,652 (30.9)	0	0	0	1605 (23.4)	0
2000–2009	9094 (47.7)	10,475 (52.5)	35,579 (32.4)	11,615 (29.5)	0	21,291 (37.3)	5708 (31.3)	2432 (36.7)	0	2537 (37.1)	826 (27.0)
≥2010	10,194 (52.2)	7517 (37.7)	27,726 (25.2)	27,726 (70.5)	195,575 (100)	12,501 (21.9)	12,501 (68.7)	4189 (63.3)	14,065 (100)	2236 (32.7)	2236 (73.0)
Years of data before diagnosis, mean (SD)	7.5 (4.3)	13.1 (6.2)	37.9 (9.3)	7.1 (2.9)	8.2 (2.3)	37.9 (8.5)	6.9 (2.9)	10.3 (4.5)	8.2 (2.2)	40.4 (8.5)	7.3 (2.9)

Sweden 1: Cases included in the analysis of health conditions; Sweden 2: Cases included in the analysis of prescribed medications. SD, standard deviation.

a Individuals who were disease-free at the time of the cases' diagnosis were randomly selected and individually matched to cases by sex and age. The years of data before the diagnosis were consistent within each case–control pair, as the data were sourced from national registers across the four countries.

### Prescribed medications before the diagnosis of AD, PD, and ALS

**Fig. 2:**
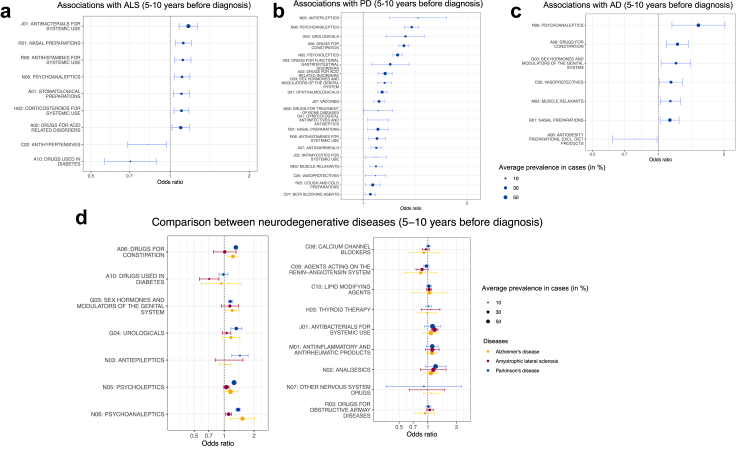
**Prescribed medications associated with the subsequent diagnosis of Alzheimer's disease, Parkinson's disease, and amyotrophic lateral sclerosis during 5–10 years before the diagnosis, meta-analyses from Australia, France, the UK, and Sweden**. AD, Alzheimer's disease; ALS, amyotrophic lateral sclerosis; PD, Parkinson's disease. Panel a: nine out of 43 medications were associated with amyotrophic lateral sclerosis 5–10 years later based on meta-analysis (n = 261,228; *P* < 0.05 from Wald test); panel b: twenty out of 45 medications were associated with Parkinson's disease 5–10 years later based on meta-analysis (n = 591,449; *P* < 0.05 from Wald test); panel c: seven out of 49 medications were associated with Alzheimer's disease 5–10 years later based on meta-analysis (n = 510,743; *P* < 0.05 from Wald test). Only classes of medications with an incidence rate ratio from the meta-analysis above 1.2 or below 0.8 and *P*-value<0.05 in at least one of three diseases are shown in panel d. The size of the dot is the average prevalence of prescribed medications in cases across countries. Bars correspond to 95% confidence intervals in the meta-analysis after correction for multiple testing.

We observed from meta-analyses that psychoanaleptics and nasal preparations were more frequently prescribed 5–10 years before the diagnosis of AD [IRR (95% CI): 1.53 (1.16–2.02) and 1.13 (1.03–1.25) respectively], PD [1.38 (1.32–1.45) and 1.10 (1.03–1.18)], and ALS [1.11 (1.04–1.19) and 1.12 (1.05–1.20)], compared with controls (*P* < 0.05 from Wald test; Fig. 2; Tables S7–10). We also found that individuals with AD and PD, respectively, compared with controls, had more prescribed use of medicines for constipation [1.22 (1.09–1.37) and 1.31 (1.27–1.36)], sex hormones and modulations of the genital system [1.20 (1.03–1.41) and 1.15 (1.10–1.21)], vasoprotectives [1.14 (1.02–1.29) and 1.09 (1.01–1.16)], and muscle relaxants [1.14 (1.02–1.27) and 1.09 (1.05–1.13)] 5–10 years before diagnosis. In contrast, medications for acid-related disorders were more frequently prescribed 5–10 years before the diagnosis of PD and ALS [1.16 (1.10–1.21) and 1.10 (1.02–1.18) respectively]. Compared to controls, patients with AD exhibited a reduced likelihood of being prescribed anti-obesity preparations during 5–10 years before diagnosis [0.78 (0.62–0.99)]. Patients with PD had a higher risk of being prescribed medications for functional gastrointestinal disorders [1.20 (1.06–1.36)], antidiarrheals [1.09 (1.05–1.13)], beta blocking agents [1.05 (1.01–1.09)], gynaecological anti-infectives and antiseptics [1.10 (1.03–1.18)], urologicals [1.33 (1.17–1.51)], antimycotics for system use [1.09 (1.02–1.17)], vaccines [1.11 (1.07–1.15)], drugs for treatment of bone diseases [1.11 (1.01–1.22)], antiepileptics [1.44 (1.19–1.74)], psycholeptics [1.26 (1.22–1.30)], cough and cold preparations [1.07 (1.01–1.12)], antihistamines for systemic use [1.10 (1.04–1.16)], and ophthalmologicals [1.14 (1.10–1.17)] during 5–10 years before diagnosis, compared to controls. In contrast, patients with ALS had a lower risk of being prescribed antidiabetics [0.71 (0.56–0.89)] and antihypertensives [0.82 (0.69–0.97)], but a higher risk of being prescribed stomatological preparations [1.10 (1.03–1.19)] and corticosteroids for system use [1.10 (1.03–1.18)], than controls. The results from the Hartung-Knapp-Sidik-Jonkman method of random effect models were largely similar (Tables S8–S10). Furthermore, similar associations were observed for the period of 2–5 years before diagnosis (Tables S7 and S11–S13). The use of prescribed medications for constipation, urologicals, antiepileptics, psycholeptics, and psychoanaleptics increased over the 10 years prior to the index date in both cases of AD, PD, and ALS and their controls; however, the increase was higher in cases compared to controls (Figure S6). The associations were similar in men and women (Tables S14–S19).

### Health conditions before the diagnosis of AD, PD, and ALS

**Fig. 3:**
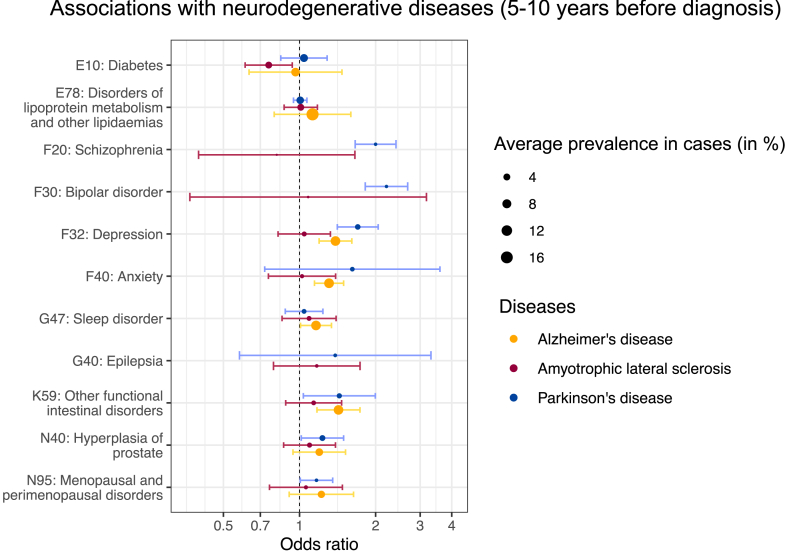
**Health conditions associated with the subsequent diagnosis of Alzheimer's disease, Parkinson's disease, and amyotrophic lateral sclerosis during 5–10 years before the diagnosis, meta-analyses from France, the UK, and Sweden**. Health conditions presented in this figure are related to the prescribed medications that were associated with the neurodegenerative diseases shown in Fig. 2 (n = 1,286,683 for Alzheimer's disease, 1,019,481 for Parkinson's disease, and 148,148 for amyotrophic lateral sclerosis; *P* < 0.05 from Wald test). The size of the dot is the average prevalence of prescribed medications in cases across countries. Bars correspond to 95% confidence intervals in the meta-analysis after correction for multiple testing.

In line with the results on psychoanaleptics, the meta-analysis showed that, compared to controls, patients with AD had a higher prevalence of depression [1.39 (1.20–1.61)], anxiety [1.31 (1.15–1.49)], and sleep disorder [1.16 (1.01–1.34)], whereas patients with PD had a higher prevalence of depression [1.70 (1.41–2.05)], schizophrenia [2.00 (1.66–2.41)], and bipolar disorder [2.21 (1.82–2.68)], during 5–10 years before diagnosis (*P* < 0.05 from Wald test; Fig. 3; Tables S20–S22). Both patients with AD and PD had an elevated prevalence of constipation during 5–10 years prior to diagnosis [1.43 (1.17–1.73) and 1.44 (1.04–1.99) respectively]. Diabetes was observed to be associated with a lower risk of ALS [0.75 (0.62–0.92)], but such an association was not found for AD [0.96 (0.63–1.47)] or PD [1.04 (0.84–1.29)] (Tables S20 and S23). In contrast, acute myocardial infarction [0.86 (0.77–0.96)] and atherosclerosis [0.79 (0.64–0.98)] were less prevalent among patients with PD, compared to controls, during 5–10 years before diagnosis (Table S22). These associations were mostly observed for the periods of 2–5 years and >10 years before diagnosis (Tables S20 and S24–S29).

### Blood biomarkers before the diagnosis of AD, PD, and ALS

A higher level of triglycerides was associated with a lower risk of AD 5–10 years later [0.69 (0.50–0.95)] (Table 2; Tables S30–S32). Similar association was found for Apolipoprotein B (ApoB) and PD [0.60 (0.37–0.97)]. Compared with controls, patients with AD had higher levels of ApoB [1.13 (1.01–1.25)], ApoB/apolipoprotein A (ApoA) ratio [1.16 (1.04–1.30)], low-density lipoprotein (LDL) [1.17 (1.02–1.35)], and LDL/high-density lipoprotein (HDL) ratio [1.13 (1.03–1.23)] measured >10 years earlier. In contrast, patients with PD had lower levels of ApoB [0.85 (0.72–1.00)], triglycerides [0.79 (0.67–0.92)], and leukocyte [0.69 (0.51–0.94)] than controls. We did not observe clear associations for ALS.

**Table 2:** Incidence rate ratios and 95% confidence intervals of Alzheimer's disease, Parkinson's disease, and amyotrophic lateral sclerosis in relation to blood biomarkers in Sweden.^a^

Biomarkers	Reference values	5–10 years before the diagnosis			>10 years before the diagnosis		
		Alzheimer's disease	Parkinson's disease	Amyotrophic lateral sclerosis	Alzheimer's disease	Parkinson's disease	Amyotrophic lateral sclerosis
Apolipoprotein A (ApoA)	Male: <2 g/L; Female: <2.3 g/L	0.28 (0.01–6.42)	1.37 (0.16–11.99)	–b	1.24 (0.69–2.22)	0.66 (0.23–1.85)	1.39 (0.12–16.13)
Apolipoprotein B (ApoB)	Male: <1.3 g/L; Female: <1.5 g/L	0.87 (0.58–1.29)	0.60 (0.37–0.97)	0.69 (0.19–2.55)	1.13 (1.01–1.25)	0.85 (0.72–1.00)	0.92 (0.59–1.45)
Ratio of ApoB/ApoA	Male: <0.8; Female: <0.7 g/L	0.83 (0.56–1.24)	0.80 (0.49–1.31)	1.41 (0.33–6.06)	1.16 (1.04–1.30)	0.91 (0.76–1.09)	1.15 (0.71–1.88)
High-density lipoprotein (HDL)	Male: <2.1 mmol/L; Female: <2.7 mmol/L	1.23 (0.55–2.75)	0.75 (0.25–2.26)	0.60 (0.06–6.21)	1.02 (0.80–1.31)	0.79 (0.55–1.15)	0.74 (0.23–2.35)
Low-density lipoprotein (LDL)	Male: <5.3 mmol/L; Female: <5.3 mmol/L	0.87 (0.53–1.42)	0.73 (0.37–1.46)	0.77 (0.16–3.71)	1.17 (1.02–1.35)	0.87 (0.67–1.12)	1.07 (0.54–2.09)
Ratio of LDL/HDL	Male: <2.5; Female: <2.5	0.93 (0.69–1.26)	0.77 (0.52–1.14)	1.08 (0.42–2.78)	1.13 (1.03–1.23)	0.93 (0.81–1.06)	1.00 (0.69–1.47)
Total cholesterol	Male: <7.8 mmol/L; Female: <7.8 mmol/L	0.98 (0.72–1.35)	0.83 (0.52–1.32)	0.74 (0.24–2.30)	1.06 (0.94–1.18)	0.88 (0.71–1.08)	1.42 (0.86–2.35)
Triglycerides	Male: <2.6 mmol/L; Female: <2.6 mmol/L	0.69 (0.50–0.95)	0.70 (0.47–1.03)	1.00 (0.47–2.10)	1.06 (0.96–1.17)	0.79 (0.67–0.92)	0.96 (0.63–1.44)
Glucose	Male: <7 mmol/L; Female: <7 mmol/L	0.83 (0.59–1.18)	0.78 (0.51–1.21)	0.59 (0.14–2.50)	1.05 (0.91–1.21)	0.97 (0.78–1.22)	0.53 (0.21–1.33)
Creatinine	Male: <105 μmol/L; Female: <90 μmol/L	0.86 (0.69–1.07)	1.04 (0.78–1.39)	1.12 (0.49–2.55)	0.92 (0.83–1.02)	1.09 (0.92–1.28)	0.96 (0.58–1.58)
Leukocyte	Male: <8.8 ∗10ˆ9/L; Female: <8.8 ∗10ˆ9/L	0.74 (0.50–1.10)	0.79 (0.45–1.40)	1.75 (0.45–6.81)	0.92 (0.77–1.09)	0.69 (0.51–0.94)	1.26 (0.54–2.94)
Haptoglobin	Male: <1.9 g/L; Female: <1.9 g/L	0.54 (0.25–1.15)	0.82 (0.30–2.21)	0.91 (0.18–4.71)	0.83 (0.64–1.08)	0.94 (0.61–1.44)	1.14 (0.34–3.78)
C-reactive protein	Male: <5 mg/L; Female: <5 mg/L	0.85 (0.66–1.09)	0.89 (0.65–1.22)	1.70 (0.82–3.52)	0.95 (0.87–1.02)	1.02 (0.90–1.15)	0.99 (0.71–1.38)

a Adjusted by inclusion of covariates (sex, country of birth, calendar year of blood sampling, calendar year of the index date, and age at the index date) in the logistic regression models.

b All cases of amyotrophic lateral sclerosis had the level of apolipoprotein A higher than 2 g/L (male) or 2.3 g/L (female) during 5–10 years before diagnosis.

## Discussion

Our large-scale multinational population-based study from Australia, France, the UK, and Sweden showed that the prescription of psychoanaleptics and nasal preparations was associated with an increased risk of AD, PD, and ALS 5–10 years later. Findings from the meta-analyses suggested that potential indications for the prescription of psychoanaleptics, e.g., depression, anxiety, sleep disorder, schizophrenia, and bipolar disorder, were associated with an elevated risk of AD and PD 5–10 years later. Overall, we identified a total of 23 groups of medications that were associated with an increased risk of at least one NDD 5–10 years following the prescription. We also found that individuals with higher-than-normal levels of ApoB, ApoB/ApoA ratio, LDL, and LDL/HDL ratio had an increased risk of AD >10 years later, whereas those with higher-than-normal levels of ApoB, triglycerides, and leukocyte had a reduced risk of PD >10 years later.

The present study comprehensively compared medical indices for NDDs, including prescribed medications, clinically diagnosed health conditions, and blood biomarkers, using population-based data from Australia, France, the UK, and Sweden. Running analyses on health registries from multiple countries mitigates biases inherent in single-country studies, e.g., specific population demographics or healthcare system. These databases typically encompass a significantly larger and more diverse patient population compared to traditional cohorts, providing a comprehensive representation of real-world healthcare scenarios.^26^ They also offer a unique advantage in their ability to provide extensive temporal coverage compared to traditional research cohorts. The data from all countries show similar average ages at the diagnosis of NDDs, consistently showing that AD is typically diagnosed at the oldest age, followed by PD and then ALS, alongside comparable sex difference in the prevalence of different NDDs. These figures are in line with previous reports.^3^^,^^27^ Further, by exhaustively testing all possible associations within large, diverse datasets, we can uncover early indicators and common risk factors that might otherwise be overlooked, ultimately contributing to more effective prevention strategies. In addition, the associations observed for prescribed medications and health conditions during the periods of 2–5 and 5–10 years prior to the diagnosis were largely consistent, further strengthening the robustness of our findings.

Our findings align with existing evidence that psychotropic medications and related health conditions are common prodromal factors for NDDs. For example, both the present study and previous research found that psychoanaleptics (predominantly antidepressants) and depression were associated with an increased risk of subsequent AD and PD a few years later (e.g., 5–10 years).^6^, ^7^, ^8^, ^9^^,^^28^^,^^29^ The availability of multiple sets of exposures (e.g., medications and heath conditions) enabled us to cross-validate and further understand the observed associations. For instance, the findings of the associations of psychoanaleptics and several psychiatric disorders with the risk of subsequent NDDs are mutually supportive. The evidence supporting a relationship between depression and the subsequent risk of ALS is relatively limited.^30^^,^^31^ The present study extends current knowledge by revealing that psychoanaleptics prescribed 5–10 years ago was associated with a higher risk of ALS. Our findings also showed associations of schizophrenia and bipolar disorders with subsequent AD (in Sweden) and PD, in line with previous studies.^32^, ^33^, ^34^, ^35^

Constipation may be an important prodromal symptom of AD and PD. Our analyses showed an elevated risk of AD and PD among individuals with constipation or who were prescribed medications for constipation 5–10 years earlier. These results echo the previously reported associations for AD and other dementias and PD.^6^^,^^8^^,^^9^^,^^29^^,^^36^ In contrast, we observed a relationship between prescription of constipation medications and the risk of ALS 5–10 years later in France but not in the meta-analysis. Further studies are required to clarify if constipation is indeed a prodromal symptom for ALS.

Our findings suggest that both the prescription of antidiabetics and the diagnosis of diabetes were associated with a reduced risk of ALS 5–10 years later. This is in line with previous findings on protective effect of type 2 diabetes identified during the decade before diagnosis on ALS risk.^37^ It is well documented that diabetes is associated with the risk of AD and PD^38^^,^^39^; however, diabetes was identified at various periods before the diagnosis of AD or PD in these studies. For instance, one study reported that diabetes diagnosed up to six years earlier was associated with a higher risk of AD.^7^ We observed associations of diabetes and medications used in diabetes with AD or PD in the Swedish data only but not in the meta-analysis of data from multiple countries. As diabetes was ascertained from hospital-based inpatient or outpatient records in the Swedish data, these associations may be limited to diabetes with complications or type 1 diabetes with which patients routinely visit specialists. More work is needed to elucidate the heterogeneity of the associations we observed between countries.

Despite growing evidence showing associations between cardiovascular diseases (e.g., hypertension and ischemic heart disease) and the risk of AD and PD,^40^^,^^41^ knowledge on whether cardiovascular diseases and their medications are prodromal factors for AD and PD remains relatively limited.^7^^,^^29^^,^^36^ Previous studies reported a higher risk of AD in relation to hypertension diagnosed up to 15 years earlier^7^ and of PD in relation to hypotension diagnosed up to 8 years earlier.^36^ Our analysis did not confirm these associations but demonstrated that patients with AD had a higher prevalence of varicose veins in the lower extremities 5–10 years earlier while patients with PD had a lower prevalence of myocardial infarction and atherosclerosis compared to controls. We found that antihypertensives use was associated with a lower risk of ALS 5–10 years later. Previous smaller studies had, on the other hand, reported mixed results.^37^ Furthermore, higher levels of ApoB, ApoB/ApoA ratio, LDL, and LDL/HDL ratio were associated with an increased risk of AD while higher levels ApoB and triglycerides were associated with a reduced risk of PD, >10 years later. Since only the latest measurement of biomarkers during the recruitment period of AMORIS was utilised in our analysis, future investigation is needed to model trajectories of biomarkers before diagnosis of NDDs and confirm if these biomarkers measured during the prodromal stage are indicative of NDDs.

Infection might be a risk factor for NDDs. Our prior research, along with findings from other studies, has indicated that both infections and the usage of antibiotics might be associated with increased risks of AD, PD, and ALS.^37^^,^^42^^,^^43^ Although the present study did not find a consistent association of either diagnoses of infection or use of anti-infectious medication with the risk of any of the studied NDDs 5–10 years later, the point estimates consistently suggested a positive trend. Additionally, our findings showed that nasal preparations were commonly associated with AD, PD, and ALS. Consistent with previous findings,^44^^,^^45^ we observed a trend showing that rhinitis, the most common indication for nasal preparations, was also associated with a higher risk of the three NDDs although the results were not statistically significant after correction for multiple comparison. The wide confidence intervals are likely attributable to the low prevalence of rhinitis in both cases and controls, identified through hospital-based data (inpatient and outpatient care) for PD and ALS in France and Sweden. Mild cases of rhinitis, often managed in primary care, were largely missed in this analysis.

There are several limitations in this study. First, the diversity of data sources has led to a varied prevalence of medications and health conditions across countries, which may have contributed to heterogeneity in the estimates. For instance, anxiety was associated with an increased risk of PD 5–10 years later in France and Sweden. However, the confidence intervals exhibited no overlap between the two countries and the meta-analysis revealed insignificant results. On the other hand, the heterogeneity in data sources helps to ensure that any observed associations are likely robust to varying populations and healthcare systems. Second, the families of medications identified by the first three digits of ATC codes are relatively broad and can correspond to multiple indicating health conditions, making it challenging to disentangle the links between specific medications and their related diseases and NDDs. While we analysed all recorded medications, for certain prescription there were only a few cases or controls exposed before diagnosis, which may introduce sparse-data bias into the estimations. To avoid misleading results, we did not report estimations for rare medications and excluded them from the meta-analysis. Third, the interpretation of the associations between blood biomarkers and the risk of NDDs should be done with caution as these biomarkers were measured at middle age (mean: 43 years) whereas most NDDs were diagnosed after age 60. Future analyses with longitudinal measures of the studied biomarkers from middle until old age are warranted to confirm our results. Fourth, we were unable to account for the number of healthcare visits prior to diagnosis in our analysis, as none of our databases included comprehensive healthcare data across all settings (primary, outpatient, and inpatient care). However, since the prescription of certain medications, such as medications for constipation, psycholeptics, and psychoanaleptics, was significantly more prevalent closer to the diagnosis of NDDs, the analysis of the 5- to 10-year period before diagnosis is unlikely to be significantly biased by this factor. Additionally, we were unable to analyse if over-the-counter medication use is associated with future diagnosis of NDDs due to the lack of such information. Furthermore, the information on potential confounders such as genetic and familial environmental factors, lifestyle factors, etc. was not available in the databases used in this study. We calculated E-values to evaluate the robustness of results regarding psychoanaleptics,^46^ which were consistently associated with AD, PD, and ALS. The E-values for the association between psychoanaleptics and ALS [IRR 1.11 (95% CI: 1.04–1.19)] were 1.46 for the point estimate and 1.24 for the lower confidence limit. This indicates that an unmeasured confounder would need to be associated with psychoanaleptics and ALS with a minimum IRR of 1.24 to account for the observed association. Given the relatively low E-values for the confidence limits, it is possible that certain unmeasured confounders may contribute to this association. Regardless, substantially stronger confounding effects would be required to account for the associations between psychoanaleptics and AD or PD.

### Conclusion

Conducting longitudinal studies across various health registries and diseases is both feasible and valuable. Our large-scale study leveraging data from four countries indicates that depression and psychoanaleptics may serve as predictors for a subsequent diagnosis of AD, PD, and ALS in 5–10 years. Additionally, our findings highlight constipation and related medication use as potential indicators for a higher subsequent risk of AD and PD, while diabetes and antidiabetics use emerge as potential indicators for a lower risk of ALS. Our findings have practical implications for the development of targeted interventions, laying the groundwork for a more integrated comprehension of the intricate prodromal phases of NDDs with the goal of influencing future research and clinical management strategies. Future studies should look at ATC sub-classes and individual drugs to identify candidates for repurposing.

## Contributors

The consortium was responsible for data access, data harmonisations, methodological supports, and regular workshops and meetings. DW, AF, OG, NRW, SD, AM, BCD, FF, and TN designed the study. SD, AM, and FF supervised the study. DW, AF, OG, and TN performed formal analysis. DW, OG, TN, and KZ prepared tables and figures. DW, TN, and AM have accessed and verified the underlying data. DW and TN wrote the first draft of the manuscript. All authors contributed to and edited the manuscript. All authors read and approved the final version of the manuscript.

## Data sharing statement

The data from the SNDS (France) are governed by Articles R. 1461–11 to R. 1461-17 of the French Public Health Code and the French data protection authority decision CNIL-2016-316, and cannot be shared publicly. Access to the CNAM data portal is restricted to the ARAMIS team through the INRIA affiliation, in compliance with the regulations.

The Australian and THIN data used in this study were obtained from the Australian Institute of Health and Welfare (linkage@aihw.gov.au) and the Cegedim company (info@the-healthimprovement-network.co.uk), respectively. The Australian data may be requested for research purposes from the Australian Institute of Health and Welfare by researchers who fulfil specific requirements, e.g., ethical approval from the Australian Institute of Health and Welfare Ethics Committee.

The Swedish data are register-based and pseudonymised, and are subject to General Data Protection Regulation (GDPR) and cannot be shared openly. Register data are possible to access for research purposes for qualified researchers upon request to the Swedish National Board of Health and Welfare and Statistics Sweden, provided that they meet specific requirements. Data from the AMORIS cohort can be made available upon request to the Research Data Office at Karolinska Institutet via rdo@ki.se after ensuring compliance with relevant legislation and GDPR.

The code for meta-analysis is available at https://github.com/thomasnedelec/meta_lemerend.

## Declaration of interests

The LeMeReND consortium was established to build a predictive algorithm for neurodegenerative diseases, designed for implementation in primary care settings. This initiative is supported by financial backing from the EU Joint Programme–Neurodegenerative Disease Research and involves researchers from Australia, France, and Sweden. NK has received consulting fee from Sobi and holds shares in AstraZeneca. KM received support from the Swedish Research Council for Health, Working Life and Welfare for research projects. KM also serves as a board member on the board for national screening of diseases at the Swedish National Board of Health and is the Vice chair in the Swedish Epidemiology Association. MF chairs the steering group for the AMORIS cohort. BV received support from the French government under the “France 2030” investment plan managed by the French National Research Agency (reference: ANR-17-EURE-0020) and from Excellence Initiative of Aix-Marseille University - A∗MIDEX. BV is also a panel member of the advisory board for the Ministry of Health in France and a member of the Haut Conseil de la Santé Publique in Paris, France. SD received support from Sanofi and Biogen for research projects. SD was compensated by the Union régionale des professionnels de santé (URPS) de Paris for lectures, holds a patent (“A method for determining the temporal progression of a biological phenomenon and associated methods and devices”; PCT/IB2017/052722), and is a shareholder of Qairnel SAS. BCD was compensated by the International Statistical Genetics Workshop for lectures and received support from AFRAN—Australia + French embassy to attend a conference. All other authors declare no competing interests.

## Appendix A Supplementary data

Supplementary Tables

Members in LeMeReND
