# Supplementary material for: Ten years preceding a diagnosis of neurodegenerative disease in Europe and Australia: medication use, health conditions, and biomarkers associated with Alzheimer's disease, Parkinson's disease, and amyotrophic lateral sclerosis
Source: eBioMedicine. 2025 Feb 5;113:105585. doi: 10.1016/j.ebiom.2025.105585 (PMC11847299; doi:10.1016/j.ebiom.2025.105585)
Supplement: Supplementary Figures [file mmc1.docx]

**Supplementary figures**

**Contents**

Figure S1. Flowchart of identification of study participants in Australia

Figure S2: Flowchart of identification of study participants for Parkinson’s disease and amyotrophic lateral sclerosis in France

Figure S3: Flowchart of identification of study participants for Alzheimer’s disease in France and the UK

Figure S4. Flowchart of identification of study participants in Sweden

Figure S5. Flowchart of identification of study participants in the Swedish AMORIS cohort

Figure S6. Average incidence rate of prescribed medications before the diagnosis across France, the UK, and Sweden

**Figure S1. Flowchart of identification of study participants in Australia.** ALS=amyotrophic lateral sclerosis.

French population with a certified identification number recorded in the SNDS: n=85,457,762

283,838 patients with PD

195,575 patients with incident PD

195,575 controls

16,927 patients with ALS

14,065 patients with incident ALS

140,650 controls

Selected one and ten controls free of any PD and ALS for each patient with PD and ALS respectively, individually matched by sex, year of birth, and French regional code of residence

Excluded 88,263 patients with PD before

Excluded 2862 patients with ALS before

Identified patients with a PD or ALS in the period 2014-2022

The analyses of prescription of drugs and health conditions associated with subsequent PD or ALS

**Figure S2. Flowchart of identification of study participants for Parkinson’s disease and amyotrophic lateral sclerosis in France.** ALS=amyotrophic lateral sclerosis; PD=Parkinson’s disease; SNDS=French National Health Data System.

UK population with at least 2 years follow-up in THIN: n=12,500,000

59,912 patients with AD in UK

The analyses of prescription of drugs and health conditions associated with subsequent AD

19,940 patients with incident AD in UK

19,940 controls

20,545 patients with AD in FR

19,056 patients with incident AD in FR

19,056 controls

Selected one control free of any neurodegenerative diseases for each patient with AD, individually matched by sex and age at last contact (±1 year)

Random down sampling + exclusion if less than two years of data before AD

Exclusion if less than two years of data before AD

French population with at least 2 years follow-up in THIN: n=8,650,000

**Figure S3: Flowchart of identification of study participants for Alzheimer’s disease in France and the UK.** AD=Alzheimer’s disease; THIN= The Health Improvement Network.

**Figure S4. Flowchart of identification of study participants in Sweden.** We selected ten controls who were free of any neurodegenerative diseases, including any dementia, Parkinson’s disease, Parkinsonism, amyotrophic lateral sclerosis, Huntington disease, and any other degenerative diseases of nervous system, prior to the index date for each patient. AD=Alzheimer’s disease; ALS=amyotrophic lateral sclerosis; NDD=neurodegenerative disease; PD=Parkinson’s disease.

**Figure S5. Flowchart of identification of study participants in the Swedish AMORIS cohort.** AMORIS= Apolipoprotein MOrtality RISsk; AD=Alzheimer’s disease; ALS=amyotrophic lateral sclerosis; PD=Parkinson’s disease.

**a.**

**b.**

**c**.

**Figure S6. Average incidence rate of prescribed medications before the diagnosis across France, the UK, and Sweden.** a. Alzheimer’s disease: France, the UK, and Sweden; b. Parkinson’s disease: France and Sweden; c. amyotrophic lateral sclerosis: France and Sweden. A06: medications for constipation; A10: drugs used in diabetes; G03: sex hormones and modulators of the genital system; G04: urological drugs; N03: antiepileptics; N05: psycholeptics; N06: psychoanaleptics.
