# Supplementary material for: Ten years preceding a diagnosis of neurodegenerative disease in Europe and Australia: medication use, health conditions, and biomarkers associated with Alzheimer's disease, Parkinson's disease, and amyotrophic lateral sclerosis
Source: eBioMedicine. 2025 Feb 5;113:105585. doi: 10.1016/j.ebiom.2025.105585 (PMC11847299; doi:10.1016/j.ebiom.2025.105585)
Supplement: Members in LeMeReND [file mmc3.docx]

**The list of members in the LeMeReND consortium**

| **First name** | **Surnames** |
| --- | --- |
| **French team** |  |
| Stanley | Durrleman |
| Bruno | Ventelou |
| Thomas | Nedelec |
| Octave | Guinebretiere |
| Karim | Zaidi |
| **Swedish team** |  |
| Fang | Fang |
| Dang | Wei |
| Fen | Yang |
| **Australian team** |  |
| Allan | McRae |
| Naomi | Wray |
| Baptiste | Couvy-Duchesne |
| Anna | Freydenzon |
| Lydie | Tran |
| Evans | Cheruiyot |
